# Factors associated with patient readmission to a specialised psychiatric hospital in the Eastern Cape

**DOI:** 10.4102/sajpsychiatry.v28i0.1878

**Published:** 2022-12-19

**Authors:** Razia Gaida, Chinedum Okafor, Lichelle Janse van Vuuren, Adlai S. Davids

**Affiliations:** 1 Centre for Community Technologies, School of Engineering, Nelson Mandela University, Gqeberha, South Africa; 2 Eastern Cape Department of Health, Gqeberha, South Africa; 3 Human and Social Capabilities, Human Sciences Research Council, Gqeberha, South Africa; 4 Faculty of Health Sciences, Nelson Mandela University, Gqeberha, South Africa

**Keywords:** psychiatry, readmission, health, Eastern Cape, South Africa

## Abstract

**Background:**

Hospital readmissions increase healthcare system costs and can place additional strain on already sparse government funds and under-resourced hospitals. Few studies have investigated readmission of patients in mental health facilities in South Africa.

**Aim:**

The study aimed to identify the factors associated with readmission of patients discharged from an acute psychiatric public hospital in South Africa.

**Setting:**

The study was conducted at an acute psychiatric public hospital.

**Method:**

A retrospective review of medical records was conducted for all patients admitted and readmitted between January 2018 and December 2019.

**Results:**

From the pool of patient records analysed (*n* = 516), 93 (18.02%) were readmitted, of which the majority (75.27%) were male. The average age of patients readmitted was 27.24 ± 11.16 years, which was significantly younger than the total sample (*p* < 0.05; CI 1.095–7.105). Findings indicated that patients who completed lower levels of education, were unemployed and were diagnosed with substance (mono- or polysubstance) use disorder (*n* = 93; 100%), schizophrenia (*n* = 33; 35.48%), bipolar disorder (*n* = 9; 9.68%) or intellectual disability (*n* = 9; 9.68%) were more frequently readmitted, with the average length of stay varying widely between patients.

**Conclusion:**

Younger patients and those living with more complex psychiatric conditions, particularly those who are substance abusers, were readmitted more frequently, indicating that these patients may require special consideration for management.

**Contribution:**

The study revealed that patients living with complex psychiatric conditions such as schizophrenia and bipolar disorder were readmitted to hospital more frequently, indicating that management of these patients at the community level is challenging.

## Introduction

Mental health disorders negatively impact the well-being of the individual and society in a variety of ways. Those affected are more likely to be unemployed,^1^ have lower education levels,^2^ face stigma,^3^ experience poor physical health with a shorter life expectancy^3,4^ and be a victim of violence.^5^ In South Africa, the number of individuals living with mental illness is widespread.^6^ The South African Stress and Health Survey showed a 30.3% lifetime prevalence for any mental disorder and a 13.3% lifetime prevalence of substance use.^7^ However, since this survey was conducted in 2003, and no follow-up surveys have been conducted, this number has likely changed.

While the concept of ‘recovery’ in the context of mental health disorders needs to be understood as a journey rather than as an endpoint,^8^ the reality is that ongoing mental health disorders have an economic impact. A report by Docrat and colleagues^9^ reported that South Africa’s expenditure on inpatient care constituted 86% of the total mental healthcare expenditure, with nearly half being spent on psychiatric hospitalisation. The report also noted that 25% of patients were readmitted to specialised psychiatric hospitals within three months of being discharged, which carried an estimated cost of $63.9 million (approximately R1 billion as of 27 June 2022), 10.4% of the total mental health expenditure for the year 2016/2017.^9^

Hospital readmissions increase public healthcare costs. In countries like South Africa and other low- and middle-income countries with limited health resources, readmissions can place an added strain on an already overburdened healthcare system.^10^ Globally, and in South Africa, factors such as age, gender, employment status, social support and education have been found to affect readmission rates, in addition to health system factors such as hospital discharge policy and linkage to community-based outpatient care.^10,11,12,13^

Although mental health is recognised as a public health crisis in South Africa, the amount of resources allocated to and policymaker concern over mental health services in South Africa is insufficient.^14^ Furthermore, studies show that the geographic location of patients – rural, suburban, township or urban areas – influences the availability and accessibility of healthcare services and can affect the quality of care received at the community level.^15,16^ Thus, these factors could conceivably influence patient readmissions. Of additional concern is the link between poverty and mental illness. Feelings of hopelessness and the higher risk of violence and poor physical health make those who live in poverty more susceptible to mental illness.^17^ Furthermore, associations between low education levels, food insecurity, inadequate housing, low social class and financial stress and mental illness have also been noted.^17,18^ There has also been conflicting evidence in various countries suggesting that income, employment and consumption may play a role in mental illness.^18^ Given the high levels of poverty in South Africa, the constant cycle of poverty and mental illness places an increased burden on mental health services in South Africa and can contribute to the ‘revolving door’ of mental healthcare admissions.^17,18^

The heading of Aim can be inserted above this paragraph readmissions to a specialised public psychiatric hospital located in the Nelson Mandela Bay Municipality in the Eastern Cape province, South Africa. The specific objectives were to examine whether sociodemographic characteristics, geographic residence, diagnoses and the medication prescribed to each patient were related to readmissions.

## Research methods and design

The study was descriptive and quantitative in nature and employed a retrospective review of medical records. The study was conducted at an acute psychiatric public hospital that serves as the main mental health service facility in the metropolitian area. The hospital has 193 beds and comprises six wards, including a small intensive care unit for acute psychiatric episodes.

The medical records of all patients admitted to the facility between January 2018 and December 2019 were anonymised and included in the study. As per facility policy, a readmission is any admission of a patient within a period of 2 years after discharge.

All medical records of patients admitted between January 2018 and December 2019 were retrospectively reviewed by a trained research assistant for the date of first admission and subsequent readmission(s). Demographic data were extracted, as well as diagnoses and medication prescribed, including psychiatric and other chronic medications. In addition, the patients’ area of residence was recorded for geographic mapping to identify potential hotspots.

The residence of the patient was geolocated using a geographical information systems (GIS) database using ArcGIS 10.5 (Esri Software, Redlands, California, United States). Data on the retrospectively reviewed medical files were captured on Microsoft Excel (Microsoft Corporation, Redmond, Washington, United States). Data were analysed using descriptive statistics. Chi-square statistics were used to examine categorical variables. *T*-tests were used to examine continuous variables. A confidence interval of 95% was used for all statistical tests, and the significant level (α) was set at 0.05. Statistical tests were conducted using Stata version 15.0 (StataCorp LLC, College Station, Texas, United States).

### Ethical considerations

The study obtained ethics approval from the Nelson Mandela University Research Ethics Committee (REC) Human (ref. no. H20-ENG-ITE-003) and the Eastern Cape Department of Health (EC_202101_009).

## Results

A total of 516 patient records were analysed between July 2021 and August 2021. The average age of the population was 31.3 ± 13.9 years (range = 13–72 years), with 45.93% of the population aged between 16 and 25 years. Male patients were on average younger than female patients at 28.5 ± 12.5 years compared to 36.6 ± 4.9 years, respectively. Most patients had completed up to a secondary level education (*n* = 244; 47.29%), and the majority were unemployed (*n* = 408; 79.07%).

**FIGURE 1:**
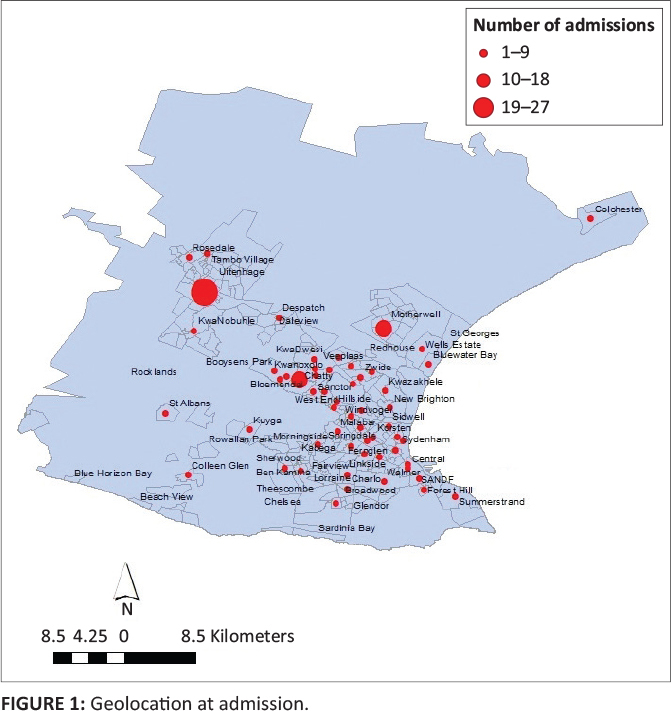
Geolocation at admission.

The data set contained 516 records, of which only 109 could be geolocated. See Figure 1. The reasons were that a vast majority of patients were either not resident in the district or that address data were incomplete. The majority of the records in the data set could be geolocated to various suburbs in the Nelson Mandela Bay health district, with slightly higher than average admission rates being seen in Bloemendal, the informal settlement area of Motherwell and the neighbouring town of Uitenhage (Kariega). Still other patients travelled from the town of Colchester, located in the north-east of the map, approximately 40 km from the study site. All patients were admitted involuntarily, which means that the patient was transferred from another hospital or admitted by police decision.

**FIGURE 2:**
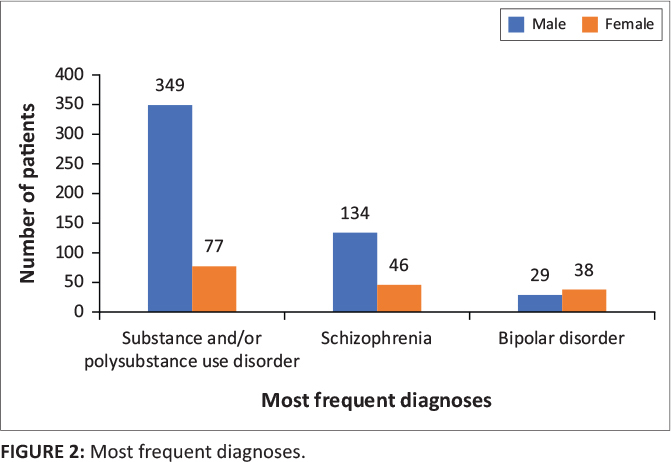
Most frequent diagnoses.

The most frequently recorded diagnoses are shown in Figure 2. The most common diagnosis was substance (mono- and polysubstance) induced psychotic episodes (*n* = 426; 82.56%) and was more common among male patients (*n* = 349; 81.9%) compared to female patients (*n* = 77; 18.1%). This was followed by schizophrenia, including schizophreniform and schizoaffective disorder, treatment-resistant schizophrenia, paranoid schizophrenia and schizophrenia with catatonia and/or fixed delusion (*n* = 180; 34.89%) and bipolar disorder, including Type 1 and Type 2 and bipolar disorder with depression and/or manic or psychotic features (*n* = 67; 12.98%). This clustering is based on diagnoses as noted in the records and not on DSM-V definitions. While 181 (35.08%) patients had just one diagnosis, 270 (52.33%) had two and the remainder (*n* = 65; 12.60%) had three diagnoses.

**FIGURE 3:**
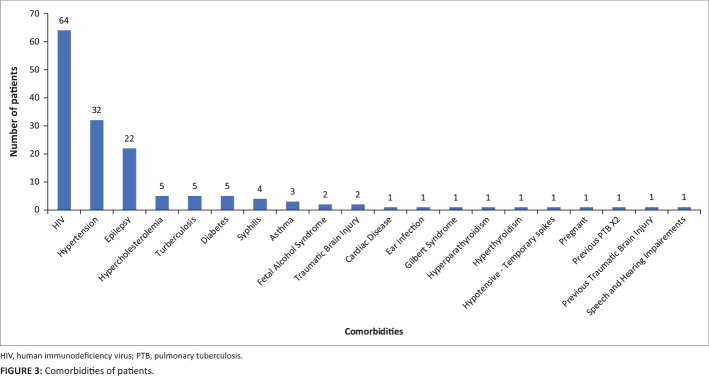
Comorbidities of patients.

Comorbidities were recorded for 167 patients (32.40%) and are summarised in Figure 3.

The three most frequently reported comorbidities were HIV (*n* = 64; 12.40%), hypertension (*n* = 32; 6.20%) and epilepsy (*n* = 22; 4.26%). An average of 1.01 ± 1.46 chronic (nonpsychiatric) medications was prescribed per patient (range = 0–11).

A total of 93 (18.02%) patients were admitted to the facility more than once between January 2018 and December 2019, of which 70 were male patients (75.27%). The average age of this group was 27.24 ± 11.16 years, which was significantly younger than the total population (*p* < 0.05; CI 1.095–7.105). There were 21 (4.07%) patients who were admitted three times during this period, three (0.58%) patients who were admitted four times and a single (0.19%) patient who was admitted seven times during the two year period. The patient who was admitted seven times was in hospital for a period of 264 days (average of 37.71 ± 29.74 days per admission) because of repeated episodes of substance-induced psychotic disorder. The demographics of the readmission population are summarised in Table 1.

**TABLE 1:** Demographics of readmissions.

Demographics	Male (*n* = 70)		Female (*n* = 23)		Total (*n* = 93)	
	*n*	%	*n*	%	*n*	%
**Age**						
16–25 years	49	52.69	9	9.68	58	62.37
26–35 years	15	16.13	3	3.23	18	19.35
36–50 years	3	3.23	6	6.45	9	9.68
51 years and older	3	3.23	5	5.38	8	8.06
**Employment status**						
Employed	6	6.45	2	2.15	8	8.06
Unemployed	60	64.52	19	20.43	79	84.95
Student	4	4.30	2	2.15	6	6.45
**Education level**						
Primary	12	12.90	2	2.15	14	15.05
Secondary	40	43.01	13	13.98	53	56.99
Matric	14	15.05	5	5.38	19	20.43
Tertiary	2	2.15	2	2.15	4	4.30
Unknown	2	2.15	1	1.08	3	3.23
**Relationship status**						
Single	57	61.29	15	16.13	72	77.42
In a relationship	8	8.60	3	3.23	11	11.83
Married	2	2.15	2	2.15	4	4.30
Divorced	3	3.23	2	2.15	5	5.38
Widow or widower	0	-	1	1.08	1	1.08

**FIGURE 4:**
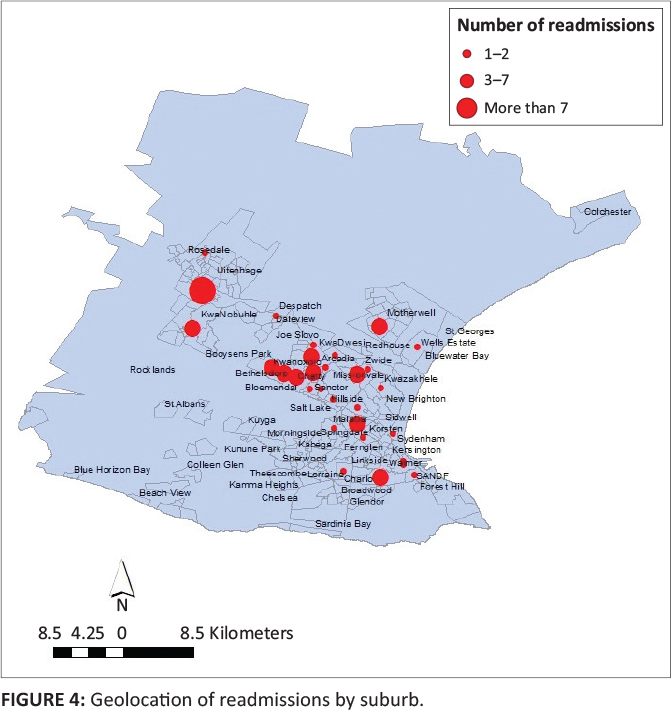
Geolocation of readmissions by suburb.

Similar to the general study sample, the majority of patients were unemployed (*n* = 79; 84.95%), single (*n* = 72; 77.42%) and had completed a secondary-level education (*n* = 53; 56.99%). While there was little difference between the average ages of male patients (27.24 ± 11.16 years) and female patients (27.01 ± 10.97 years) who experienced more than one admission in two years, a majority (62.36%) of readmitted patients were between the ages of 16 and 25 years. The suburbs from which patients were readmitted are shown in Figure 4.

There were, on average, 2.5 readmissions per suburb. Higher than the average number of readmissions were seen from the suburbs of Motherwell, KwaDwesi, Zwide, Booysens Park, Bethelsdorp, Arcadia, Gelvandale, Walmer, Bloemendal and the town of Uitenhage (Kariega) and its surrounding township KwaNobuhle. Table 2 shows the number of readmissions per suburb.

**TABLE 2:** Number of readmissions per suburb.

Suburb	Number of readmissions
Motherwell	3
KwaDwesi	3
Zwide	3
Booysens Park	3
Bethelsdorp	3
Arcadia	3
Gelvandale	3
Walmer	4
Bloemendal	5
Uitenhage (Kariega) and KwaNobuhle	22

Readmitted patients were prescribed an average of 2.69 ± 1.33 medications (psychiatric medication and chronic (nonpsychiatric) medication) compared to 2.91 ± 1.66 of the overall population (*p* > 0.05). There were just 22 patients who had recorded comorbidities, which included HIV (*n* = 11), hypertension (*n* = 4), diabetes type 2 (*n* = 2), epilepsy (*n* = 1), Gilbert syndrome (*n* = 1), traumatic brain injury (*n* = 1), tuberculosis (*n* = 1) and tuberculosis lymphadenopathy (*n* = 1). The most frequent diagnoses noted among patients who were readmitted were substance (mono- or polysubstance) use disorder (*n* = 94); followed by schizophrenia (*n* = 33), including schizophreniform disorder, paranoid schizophrenia and schizophrenia with fixed delusion and/or catatonia; and bipolar disorder (*n* = 9), including Type 1, Type 2 and bipolar disorder with manic features and intellectual disability (*n* = 9).

The length of hospital stays varied greatly, with a range of 13–147 days for first admissions (average length of stay 61.70 ± 32.66 days) compared to a second admission where the range was 3–309 days (average length of stay 65.85 ± 52.53 days). Of the 23 patients who were admitted three times, the length of stay ranged from five to 123 days (average length of stay 45.61 ± 34.11 days) while the three patients with four admissions showed a smaller range from 26 to 41 days (average length of stay 34 ± 7.56 days).

## Discussion

The readmission rate found in this study was 18.02% (*n* = 93). Readmission rates vary greatly between countries and settings. While there is a paucity of studies surrounding readmissions, studies in China have found readmission rates of 30% in Beijing,^19^ while in Guangzhou,^20^ 13.8% of patients were readmitted within 1 year of discharge. In India, a 27.2% readmission rate was found, with female patients, high poverty levels and a high education status being positively associated with readmission.^21^ In the Western Cape, a study focusing on adolescents found a 36% readmission rate over 1 year.^22^

Readmitted patients were younger than the total population and were diagnosed with substance use with complex psychiatric conditions on the schizophrenia or bipolar disorder spectrum. Similar to other global and South African studies,^11,12,13^ readmitted patients were younger, single and admitted involuntarily. Pieterse and colleagues^22^ noted that more than half of the adolescents included in the study were diagnosed with a disorder along the schizophrenia spectrum, but no association was found between diagnosis and risk of readmission.

While mental health statistics are available for the population covered by medical insurance, statistics in public sector are unavailable.^23^ A 24 h substance abuse hotline, run by the South African Depression and Anxiety Group (SADAG), noted that 63% of callers were under the age of 40 years and the majority were female (*n* = 58%).^24^ About half of these callers reported that they were depressed, while 11.5% reported a combination of depression and generalised anxiety, 7.7% reported bipolar disorder, a further 7.7% reported attention deficit hyperactivity disorder (ADHD), 3.8% reported post-traumatic stress disorder (PTSD) and 3.5% reported schizophrenia.^24^ The majority of the study population were diagnosed with substance use disorders, schizophrenia and bipolar disorder.^24^ These were the same groups readmitted most frequently, indicating that substance abuse is an ongoing problem in the Eastern Cape and that complex psychiatric conditions are not effectively managed at a primary healthcare level. In addition to other challenges, South Africa is understaffed in terms of mental healthcare practitioners, with 7.5 psychiatric nurses, 0.28 psychiatrists, 0.4 social workers and 0.32 psychologists per 100 000 population.^25^

Almost two-thirds of the study population were receiving antiretroviral treatment (ART); human immunodeficiency virus HIV infection may result in neuropsychiatric disorders, either due to social factors related to diagnosis such as stigma or the resultant impact of the diagnosis on quality of life and relationships.^26^ In advanced or uncontrolled disease, the virus, being able to cross the blood-brain barrier, may trigger a neurotoxic cascade in the central nervous system, manifesting as impaired concentration, mental slowing, slowed movements, incoordination and irritability or personality change.^27^ Other noted comorbidities such as hypertension, epilepsy and hypercholesterolaemia add to the daily pill burden and increase the risk of adverse effects due to drug–drug interactions, which may impact adherence.

Upon discharge from the study site, patients are referred to one of 18 decentralised psychiatric clinics where they may collect their medication on a monthly basis. However, geographic location has been shown to influence the accessibility and quality of healthcare received.^15,16^ In this study, more patients were readmitted from informal settlements, areas known for gang violence and drug use, and neighbouring towns where local psychiatric services are lacking. Patients who reside in Uitenhage (Kariega) do not have access to adequate local public-sector psychiatric services and no dedicated hospital. Given the large number of readmissions from Uitenhage (Kariega), this motivates for the availability of local psychiatric services to allow patients more accessible care which may result in better management. The areas of Motherwell, KwaDwesi, Zwide and Walmer are all large informal settlements, while Gelvandale, Arcadia, Bloemendal, Booysens Park and Bethelsdorp are notorious for gang violence and drug use. Neighbourhoods with poor infrastructure and high levels of crime can trigger a variety of mental health disorders including depression, anxiety, substance abuse among adults and children and psychosis, particularly in younger people aged 10–20 years.^28^ In children and adolescents, community violence, including gang violence, has been associated with substance abuse, depression, anxiety and PTSD.^28^ Discharging patients into environments with no community-level rehabilitation and limited support and healthcare facilities is not conducive to effective patient management.

Substance use causes a significant burden on individual productivity, the economy and the social aspects of individuals, as well as families and communities.^29^ Approximately 13.3% of South Africans were found to have used drugs in their lifetime.^30^ The role of environment cannot be discounted in the case of substance use, including the level of urbanisation and socio-economic status.^30^ Given the social challenges such as severe poverty and high levels of inequality in South Africa, effective surveillance and evidence-based interventions focused on substance use need to be implemented in communities with collaboration between health, police and social development services. Studies have shown that young, unemployed men living in rural areas are factors strongly associated with substance use.^29^ Similarly, the current study has shown that substance use was prevalent among young male patients and, given the readmissions, is likely an ongoing challenge in communities.

Schizophrenia is a severe mental disorder that is associated with significant disability and can affect educational and occupational performance, likely due to the early onset of the disorder, its chronic course, limited mental health resources and social stigma.^31^ Patients living with bipolar disorder present a similar picture.^32^ To reduce the morbidity associated with schizophrenia and bipolar disorder, family-focused community-based interventions to increase awareness, reduce stigma and improve adherence to medication should be implemented through the integration of services provided at the primary healthcare level.^31,32,33^

## Conclusion

While the general readmission rate was not shown to be very high, the study revealed that patients living with complex psychiatric conditions such as schizophrenia and bipolar disorder were readmitted to hospital more frequently, indicating that management of these patients at the community level is challenging. Substance use, prevalent among young male patients, was the most frequent diagnosis in both the total study population as well as patients who were readmitted, which is representative of an ongoing challenge in communities across the Nelson Mandela Bay district.

Reasons as to why these groups of patients are readmitted more frequently need to be explored. Interventions focused at community-level to empower primary healthcare staff with the knowledge, skills and support to manage patients with complex psychiatric conditions and substance use disorders need to be explored and evaluated. These interventions require interprofessional collaboration due to the multifaceted nature of the factors associated with substance use.
